# Dietary Calcium Intake in Patients Evaluated for Bone Fragility, with a Focus on Women Receiving Adjuvant Endocrine Therapy for Breast Cancer: A Single-Center Cross-Sectional Study

**DOI:** 10.3390/nu18193239

**Published:** 2026-10-01

**Authors:** Francesca Firmani, Alessandro Ciarloni, Sofia Sabatelli, Claudio Gianfelice, Gianmaria Salvio, Veronica Agostinelli, Rossana Berardi, Giancarlo Balercia, Gilberta Giacchetti

**Affiliations:** 1 Division of Endocrinology, Department of Clinical and Molecular Sciences, Polytechnic University of Marche, Azienda Ospedaliero-Universitaria delle Marche, 60126 Ancona, Italy; firmanifrancesca1@gmail.com (F.F.); s.sabatelli@pm.univpm.it (S.S.); claudio.gianfelice@ospedaliriuniti.marche.it (C.G.); g.salvio@staff.univpm.it (G.S.); g.balercia@staff.univpm.it (G.B.); gilberta.giacchetti@ospedaliriuniti.marche.it (G.G.); 2 Division of Oncology, Department of Clinical and Molecular Sciences, Polytechnic University of Marche, Azienda Ospedaliero-Universitaria delle Marche, 60126 Ancona, Italy; veroagostinelli@gmail.com (V.A.); rossana.berardi@ospedaliriuniti.marche.it (R.B.)

**Keywords:** dietary calcium intake, secondary osteoporosis, fragility fracture, breast cancer, adjuvant endocrine therapy

## Abstract

**Background/Objectives**: Adequate dietary calcium intake is essential for bone health. Despite this, calcium intake is still insufficient in many countries of the world. The study aims to provide information about dietary calcium intake of specific populations with bone fragility, with a particular focus on patients at risk of secondary osteoporosis due to adjuvant endocrine therapy (AET) for breast cancer (BC). **Methods**: Data from 132 patients referred for the first time to the Osteoporosis and Bone Metabolism Diseases outpatient clinic of the Division of Endocrinology of the Azienda Ospedaliero-Universitaria delle Marche (Ancona, Italy) were collected. Dietary calcium intake was assessed with a 7-day food frequency questionnaire; biochemical and densitometric parameters, osteoporosis etiology, and previous fragility fractures were also reported. **Results**: Patients were predominantly postmenopausal women, with severe osteoporosis (43.2%) or at risk of secondary osteoporosis (40.9%); 64.8% of patients evaluated for secondary osteoporosis were women on AET for BC. Total mean dietary calcium intake was 959 ± 396 mg/day. A lower intake was observed in younger patients with postmenopausal osteoporosis (848 ± 471 mg/day, *p* = 0.03). Patients with normal bone mineral density (631 ± 131 mg/day), without fragility fractures (906 ± 361), and evaluated for secondary osteoporosis (900 ± 364 mg/day) tended to have lower mean dietary calcium intake although without reaching statistical significance. Mean calcium intake was also lower than the recommended threshold in women on AET for BC (854 ± 345 mg/day). **Conclusions**: Patients with bone fragility should be more encouraged to increase their dietary calcium intake. Attention must be paid to women with BC on AET, who have high fracture risk and should not be discouraged a priori from consuming selected dairy products (low-fat and fermented ones) and alternative calcium dietary sources.

## 1. Introduction

Calcium intake strongly affects the development of peak bone mass in adolescents and young adults and the preservation of bone mass in adults and the elderly [1]. Adequate calcium intake is essential for maintaining proper bone health [2], and it is mainly absorbed in the small intestine with a vitamin D-dependent mechanism [3,4]. Milk and dairy products are the main dietary source of calcium, followed by vegetables, seafood products, dried fruits, cereals, almond milk, rice milk, and calcium-rich waters [5,6]. Daily calcium requirement varies during life, and according to the Italian Society for Osteoporosis, Mineral Metabolism and Skeletal Diseases (SIOMMMS) guidelines, it should be 1000 mg/day in postmenopausal women on hormone replacement treatment and in men aged 50 to 65 and 1200 mg/day in postmenopausal women not on hormone replacement treatment and in men over the age of 65 [7,8]. However, the recommended calcium intake thresholds vary worldwide, and a systematic review in 2017 showed that calcium intake is low in many countries of the world [1]. A low calcium intake negatively affects bone health, causing secondary hyperparathyroidism and an increased risk of osteoporosis [9]. Conversely, an adequate dietary calcium intake may be associated with an improvement in bone mineral density (BMD) and a reduction in the risk of fragility fractures [10,11,12,13]. However, studies conducted on this topic usually do not consider the different etiologies of osteoporosis, excluding patients with secondary osteoporosis and/or a history of carcinoma. Therefore, we have limited knowledge about the dietary calcium intake of these categories of patients, who are nonetheless at high risk of fracture. Today, breast cancer (BC) is the most frequent cause of cancer death in women, thus constituting a worldwide public health concern [14]. Women with estrogen receptor (ER)-positive BC often undergo adjuvant endocrine therapy (AET) with drugs that reduce estrogen synthesis or modulate estrogen function at the receptor level [15]. Although this therapy has significantly improved BC survival [16], it is associated with an increased risk of osteoporosis and fragility fractures [17,18]. Indeed, estrogens play an important role in maintaining bone homeostasis, stimulating bone apposition through osteoblastic activity, and inhibiting its resorption by limiting osteoclastic activity [19]. In certain cases, adjuvant endocrine therapy can be extended up to 10 years to reduce the risk of recurrence, as demonstrated recently, further increasing bone-detrimental effects [20]. Moreover, these women are frequently advised against excessive intake of dairy products, as some studies have shown an association with an increased risk of BC [21,22]. In any case, this topic is still much debated, as a recent study reported no association between dairy product intake and BC risk [23]; indeed, a meta-analysis of observational studies found that specific dairy products can reduce BC risk in both pre- and postmenopausal women [24]. Despite this, BC patients have low nutrient intake from foods, including calcium [25,26]. Studies conducted on women undergoing AET often did not consider dietary calcium intake as a primary endpoint, and a comparison with other conditions potentially impairing bone health is usually absent [27,28]. The objective of this monocentric cross-sectional study was to assess, in a cohort of patients referred for the first time to the “Osteoporosis and Bone Metabolism Diseases” outpatient clinic of our Division of Endocrinology (Azienda Ospedaliero-Universitaria delle Marche, Ancona, Italy), dietary calcium intake and its variability in patients with different clinical features (age, gender, diagnosis and etiology of osteoporosis, presence of fragility fractures, pharmacological treatment) and densitometric parameters [BMD, trabecular bone score (TBS)], with a special focus on specific subpopulations, such as women with BC on AET at risk of secondary osteoporosis. It is important to assess calcium intake in this specific population, bearing in mind that conflicting evidence in the literature may have led to a lack of awareness regarding the importance of an adequate dietary intake of calcium for bone health.

## 2. Materials and Methods

### 2.1. Population

Data derived from 132 consecutive patients (122 women and 10 men, mean age 65 ± 10 years), referred for the first time to the “Osteoporosis and Bone Metabolism Diseases” outpatient clinic of the Division of Endocrinology of the Azienda Ospedaliero-Universitaria delle Marche (Ancona, Italy) from January 2020 to December 2021, were collected. Body mass index (BMI), menopausal status, medical history (fragility fractures and presence of comorbidities potentially influencing bone metabolism, such as hypercortisolism, diabetes mellitus, neoplasia, rheumatoid arthritis, primary hyperparathyroidism, and malabsorption syndromes), and pharmacological treatments, mainly focusing on antiresorptive and anabolic drugs, were recorded. Patients were then divided into 3 groups: postmenopausal, including women with spontaneous menopause, without a history of fragility fracture; secondary osteoporosis, including subjects with well-defined clinical disorders (comprising BC on AET) potentially causing bone fragility even without an already confirmed diagnosis of osteoporosis or osteopenia; severe osteoporosis, including patients with at least one fragility fracture and without risk factors for secondary osteoporosis. Written informed consent was obtained from all individual participants included in the study. Exclusion criteria were the inability to express informed consent or to complete the food frequency questionnaire.

### 2.2. Diagnosis of Fractures

Vertebral fractures were diagnosed using quantitative vertebral morphometry performed by DXA or morphometric radiography of the thoracolumbar spine. The diagnosis of non-vertebral fractures was made considering anamnesis and/or previous instrumental evaluations. Fragility fracture was defined as a fracture manifesting without a history of trauma of enough severity to cause its occurrence. Minor osteoporotic fractures were defined as those not affecting the vertebrae, the femur, the forearm, or the proximal humerus [29] and included, in the population analyzed, fractures of the pelvis, ribs, clavicle, tibia, and fibula. No fractures of the forearm or the proximal humerus were detected in the population.

### 2.3. Food Frequency Questionnaire

During the first endocrinological evaluation, each patient completed a 7-day food frequency questionnaire derived from the International Osteoporosis Foundation (IOF) Calcium Calculator (CALCIUM—Calcium Calculator|International Osteoporosis Foundation) and validated by SIOMMMS [30], in which they were asked to indicate the number of portions of calcium-rich foods usually consumed during a meal during a typical week. These data were used to estimate daily calcium intake (mg/day). The questionnaire was administered by the investigators, and the portions were measured using household measuring units (glasses, slices, cups).

### 2.4. Flow-Chart of the Assessments

During the first endocrinological assessment at the Division of Endocrinology (Azienda Ospedaliero-Universitaria delle Marche, Ancona, Italy), the following assessments were carried out in sequence: 1. administration of the questionnaire; 2. DXA scan with concurrent DXA morphometry; 3. clinical assessment involving evaluation of DXA results and the eventual prescription of biochemical tests to investigate bone metabolism. Data regarding previously performed DXA and/or morphometric radiographies of the thoracolumbar spine were recorded to define an already reported diagnosis of osteoporosis. Ongoing pharmacological treatments for osteoporosis were also collected.

### 2.5. Biochemical Parameters

When available, the following bone metabolism biochemical parameters were collected: serum calcium and phosphorus, parathyroid hormone (PTH), bone alkaline phosphatase (bALP), 25-OH-cholecalciferol (25(OH)D) and C-terminal telopeptide of type 1 collagen (CTX). 25(OH)D, PTH, and bALP were assessed with a chemiluminescence immunoassay (CLIA) method (LIAISON^®^, DiaSorin S.p.a., Vercelli, Italy), and CTX using an electrochemiluminescence immunoassay (ECLIA) method (COBAS^®^ e601 module, Roche Diagnostics International AG, Rotkreuz, Switzerland). Normal reference ranges were the following: PTH 14–85 pg/mL and bALP 4–21 mcg/L. The normal values for CTX were <570 pg/mL for premenopausal women; <1000 pg/mL for postmenopausal women; <700 for men aged from 50 to 70 years; <850 pg/mL for men aged more than 70 years.

### 2.6. Dual-Energy X-Ray Absorptiometry

BMD was assessed with Dual-energy X-Ray Absorptiometry (DXA, Lunar Prodigy©, GE Healthcare©, Madison, WI, USA) at the proximal femur and the lumbar spine and expressed as T-score and standard deviation (SD). Because the patients enrolled were postmenopausal women, and men were all aged over 50 years, a diagnosis of osteoporosis was made in the presence of a T-score ≤ −2.5 SD at any site, while a diagnosis of osteopenia was made in the presence of a T-score between −1 and −2.5 SD at any site. Bone microarchitecture was investigated through the same DXA scans of the lumbar spine using the TBS iNsight© software (version 2.1.0.0, Med-Imaps©, Pessac, France). TBS values > 1.35, between 1.2 and 1.35, and <1.2 were indicative of normal, partially degraded, and degraded bone microarchitecture, respectively. The presence of vertebral fractures was investigated through the Vertebral Fracture Assessment (VFA).

### 2.7. Statistical Analysis

Statistical analysis was performed with IBM SPSS Statistics^®^ software, version 20.0. For continuous variables, the normality of distribution was assessed through the Shapiro–Wilk test. As all continuous variables had normal distribution, they were expressed as means ± standard deviation (SD). Categorical variables were expressed as frequencies and percentages. Differences between groups were assessed by Student’s *t*-test (in cases of two groups) and one-way ANOVA (in cases of three or more groups; the Bonferroni test was used for post hoc analysis in cases of statistically significant findings). The chi-squared test and Fisher’s exact test were used for the comparison between non-continuous variables, as appropriate. A *p* value < 0.05 was considered statistically significant.

## 3. Results

### 3.1. General Population

Table 1 summarizes the characteristics of the study population. One hundred and seventy-eight patients were consecutively screened. Forty-six refused to complete the informed consent form and/or the food frequency questionnaire; the remaining 132 patients were included in the final analysis. Patients were mainly women (n = 122, 92.4%). All the women were on spontaneous menopause or ovarian suppression due to AET. No woman was on hormone replacement therapy. All men were over 50 years old. Mean age was 65 ± 10 years. Patients not taking antiresorptive or anabolic therapy were the most representative group (41.7%), followed by those treated with bisphosphonates (36.4%). Mean dietary calcium intake in the whole population was 959 ± 396 mg/day. Ninety-five women (77.9%) and 6 men (60%) had mean daily dietary calcium intake below the recommended thresholds (≥1200 mg/die in postmenopausal women or those undergoing AET; ≥1000 mg/die in men between 50 and 65 years old; and ≥1200 mg/die in men ≥ 65 years old [7,8]). Only 19 subjects (14.4%) were taking oral calcium supplements; the doses of supplementation were not reported, so the total calcium intake was not calculated. No statistically significant differences were found between males and females in age, BMI, prevalence of fragility fractures, treatment, and dietary calcium intake. Males took oral calcium supplements more frequently than females (40% vs. 12.3%, *p* = 0.04). A large part of the population (84.1%) fell within secondary and severe osteoporosis groups (Table 1). In the secondary osteoporosis group, more than half of patients (n = 35, 64.8%) had a history of BC on AET; the remaining cases were sustained by other conditions (glucocorticoid therapy, primary hyperparathyroidism, rheumatoid arthritis, malabsorption, kidney transplant) in similar proportions. In the general population, mean values of femoral and vertebral T-score were in the osteopenia range, but the TBS was indicative of degraded bone quality both in males and females. Femoral BMD, both at the neck and total hip, was significantly lower in females, but this difference did not emerge for vertebral BMD. Bone metabolism biochemical parameters were within the normal range, without differences between males and females.

### 3.2. Subgroup Analysis

**Figure 1:**
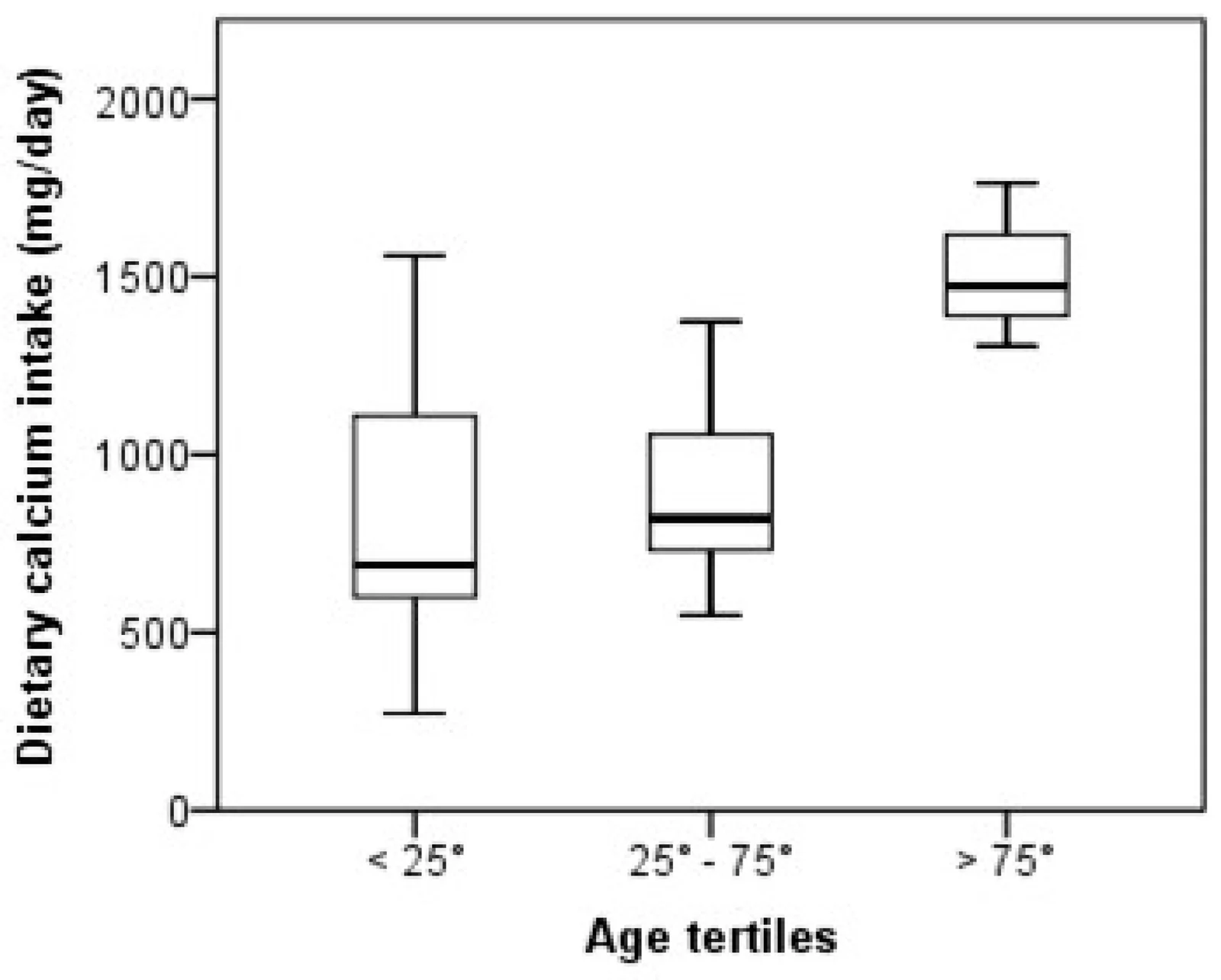
Daily dietary calcium intake in the postmenopausal group, stratified by age tertiles. Definition of age tertiles: 1st = 43–57 years (n = 8); 2nd = 58–74 years (n = 10); 3rd = 75–90 years (n = 3). The difference between groups was statistically significant (*p* = 0.03).

Considering the female population, dietary calcium intake was significantly higher in subjects on teriparatide treatment (Table 2). Moreover, dietary calcium intake tended to be higher in patients with an already reported diagnosis of osteoporosis, affected by severe osteoporosis, and with T-score < −2.5 SD, degraded TBS, and hip fractures, although not reaching statistical significance. When the female population was divided into age tertiles, daily dietary calcium intake increased with age. This difference was statistically significant in women with postmenopausal osteoporosis, as shown in Figure 1.

### 3.3. Breast Cancer Subgroup

Patients with BC were about a quarter (n = 35, 26.5%) of the entire population and more than half (64.8%) of the secondary osteoporosis group. They all underwent surgical treatment and were on AET. Nineteen (54.3%) were on aromatase inhibitors (AIs), three (8.6%) on estrogen receptor modulators (SERMs), and five (14.3%) on combined therapy with gonadotropin-releasing hormone (GnRH) agonists and AIs or SERMs. The subclass of the remaining eight (22.9%) AETs was not recorded. Furthermore, 11 (31.4%) patients had undergone chemotherapy, and 9 (25.7%) had undergone radiotherapy. All had altered bone mineralization; 7 (20%) had osteoporosis and 28 (80%) had osteopenia. Moreover, nine (25.7%) of them already reported a previous fragility fracture at first observation (Table 3). Mean dietary calcium intake was 854 ± 345 mg/day. When compared with the postmenopausal group and the severe osteoporosis group, women with breast cancer did not differ in mean dietary calcium intake, BMI, laboratory, and densitometric parameters. Twenty-seven (77.1%) women in the BC group, 13 (61.9%) in the postmenopausal group, and 42 (79.3%) in the severe osteoporosis group had daily dietary calcium intake below the recommended thresholds [7,8]. Women with BC were younger (*p* between groups < 0.005) and were less likely to be treated with oral calcium supplements. As expected, patients with severe osteoporosis had a greater frequency of fractures and treatment with anti-osteoporotic drugs, especially denosumab and teriparatide. As shown in Table 4, dietary calcium intake among the BC subgroup did not differ by age tertile, osteoporosis status, lumbar and femoral BMD and T-score, TBS, fracture status, or pharmacological treatment.

## 4. Discussion

The study focused on a cohort of patients referred for the first time in a single center for an endocrinological evaluation for the assessment of bone metabolism and fragility fracture risk. In fact, at the time of the observation, the most representative group was the one not taking antiresorptive or anabolic therapies. Mean dietary calcium intake was inadequate, according to the SIOMMMS guidelines, in the entire study population and in the postmenopausal, secondary osteoporosis, and severe osteoporosis groups. This is in line with what was reported in a review published in 2017, which revealed an inadequate dietary calcium intake in most of the world’s geographical areas, including Italy, with a mean dietary calcium intake of 765 mg/day [1]. In 2021, a multicenter Italian study found that individuals evaluated for low bone mineral density had low dietary calcium intake [30]; meanwhile, more recently, a monocentric study showed that males had higher daily calcium intake than females, and insufficient calcium intake remains widespread in the Italian population, especially among subjects with low BMD and a history of fractures [31]. This shows how, over the past years, the situation has changed very little.

In the present study, dietary calcium intake was higher in older patients; this difference was particularly evident in the postmenopausal group (Figure 1). Moreover, dietary calcium intake was higher in subjects with a diagnosis of osteoporosis. This difference reached statistical significance in those on teriparatide treatment. Patients with prior hip and minor fractures tended to have higher dietary calcium intake than those with vertebral fractures or without fractures, although the differences did not reach statistical significance. It is possible that these categories of patients had a higher dietary calcium intake because of their greater sensitization and awareness of the disease. Indeed, it must be considered that the few patients treated with injectable therapy had already visited other specialized centers and had a history of osteoporosis and multiple fractures. Therefore, presumably, they had already received dietary advice regarding calcium intake. Similarly, those who have experienced minor and hip fractures in the past may have paid more attention to calcium intake in their diet, due to the greater awareness of their condition. In fact, vertebral fractures are often misunderstood, sometimes being even asymptomatic. On the contrary, a femoral fracture (which is linked, especially in the elderly population, to very high morbidity and mortality [32] and to a significant reduction in the quality of life [33]) and even minor fractures, such as wrist fractures, are frankly perceived by the patient as a pathological event and therefore could generate greater concern and awareness of their underlying condition. Finally, older postmenopausal women may have received more education on the topic as they constitute a well-known higher risk population for developing osteoporosis and fragility fractures.

An important finding is that dietary calcium intake was very low in patients at risk of secondary osteoporosis, especially in women with breast cancer on AET. There are few studies on dietary nutrient intake in patients suffering from neoplastic diseases. Most of the works show that cancer patients have micro- and macronutrient intake below the recommended values [34,35]; this contributes to increasing the risk of developing chronic complications and worsening the outcome and life expectancy of these patients [36]. In 2020, a study found that micro- and macronutrient dietary intake of neoplastic patients, mostly affected by breast cancer, was inadequate and significantly lower than healthy subjects [25]. Other recent studies on dietary nutrient intake in women with BC showed that both BC survivors and women with BC in AET do not meet recommended nutritional guidelines [26,37]. What emerges from the literature is therefore in line with the findings described in the paper.

AET is used in hormone-sensitive BC to reduce estradiol levels (aromatase inhibitors—AIs, GnRH agonists) or prevent its local action in breast tissue (selective estrogen receptor modulators—SERMs), therefore reducing risk of tumor recurrence and increasing overall survival [16,38,39,40]. Despite the beneficial effects on neoplastic pathology, AET is associated with detrimental effects on bone tissue, with increased risk of developing osteoporosis and fragility fractures. An annual bone loss of 2–6% was observed in postmenopausal women treated with AIs, and this rate rises to over 7% in premenopausal women receiving GnRH agonists and AIs [41]. Although they have an increased fracture risk, women with BC are often advised against taking dairy products due to their presumed stimulatory effect on carcinogenesis. Indeed, studies on this topic remain inconsistent, as dairy consumption has been associated both positively [21,42] and inversely [43,44,45] with BC risk, and some authors did not find a correlation [22,23,46]. A recent review analyzed the interaction between nutrition and AET in patients with BC. The review addressed the controversy over the use of foods with potential estrogen-like effects that could compete with the anti-estrogenic action of AET. Caution is advised when consuming foods such as milk, as it may decrease the effectiveness of the therapy. However, the review notes that data are often derived from in vitro or animal model studies and need clinical validation in humans [47]. Dairy products have a complex nutritional composition: saturated fats, contaminants (pesticides, estrogen metabolites), and growth factors like insulin-like growth factor-1 (IGF-1) are associated with an increased BC risk [48,49], while calcium, vitamin D, beneficial bacteria, and conjugated linoleic acid can influence cell proliferation and differentiation, thus inhibiting tumor growth [49]. In animal studies, a high calcium intake was found to reduce the proliferation of epithelial and mammary gland cells, and to decrease the incidence of mammary tumors [50]. A recent meta-analysis [24] showed a reduction in BC risk by fermented dairy products in the postmenopausal population, and by low-fat dairy products in the premenopausal population. Another recent meta-analysis examined the association between dietary and total calcium intake and BC risk, analyzing data from seven prospective cohort studies including more than 1.5 million participants. The data suggest, although with a low quality of evidence, that a calcium intake greater than 500 mg/day resulting mainly from diet may have a protective role in the risk of breast carcinogenesis [51]. What emerges from the study is that despite their bone fragility, women with BC on AET—all of whom had an alteration of BMD and 25.7% of whom had a history of fragility fracture—have a dietary calcium intake well below the recommended national thresholds. In addition, a very small percentage (2.9%) received oral calcium supplementation. Given the results of the present study, confirming the inadequate food calcium intake, and the conflicting evidence regarding the interaction between dairy products, AET, and breast cancer, women on AET should not be discouraged a priori from ensuring an adequate calcium intake.

The study has some limitations, such as the cross-sectional design, the low sample size, and the fact that laboratory parameters were not available for all patients. In particular, the cross-sectional design cannot determine whether greater calcium intake preceded osteoporosis awareness or resulted from counseling after diagnosis or fracture. The small number of patients in each subgroup analyzed reduces the statistical power of the multiple comparisons carried out. Indeed, multivariate analysis was not carried out, and the correlations found must be considered hypothesis-generating. Furthermore, the missing data regarding calcium supplementation doses do not allow for calculation of total calcium intake. Data regarding the doses of vitamin D supplementation were also not reported. Although the questionnaire listed sources of calcium from dairy and non-dairy products, we have data on total weekly calcium intake rather than on intake from individual categories. The questionnaire does not specify which foods are low or high in fat. In addition, some potential sources of calcium are missing from the list of foods considered, such as calcium-rich waters. Recall error, seasonal variation in dietary calcium absorption, and the absence of total energy adjustment are limitations of the study. Data regarding time since breast cancer diagnosis, AET treatment duration, glucocorticoid exposure, cancer stage, or recurrence status were not available for the BC group. Furthermore, the generalizability of results is limited by the single-center design of the study. The main point of strength is the stratification of calcium intake for many different parameters, including severity of BMD alterations, presence of fragility fractures, and osteoporosis etiology, with a focus on women with BC undergoing AET. Indeed, even if other studies evaluated dietary calcium intake in BC patients treated with AET, a comparison with other populations was often absent [26,27,28]. In future studies, it will certainly be important to assess the reasons behind the observed calcium intake, to gain a better understanding of where awareness-raising initiatives on the importance of an adequate dietary intake of calcium might be most effective. Multicentric prospective studies are also surely needed to eventually confirm our findings in more heterogeneous populations.

## 5. Conclusions

Our study showed that dietary calcium intake is below the recommended Italian thresholds in a cohort of patients at high risk for osteoporosis and fragility fractures, so it is essential to counsel patients on the main dietary sources of calcium from the first medical examination. Greater attention should be paid to younger individuals, those with less compromised bone densitometric parameters, and those at risk of secondary osteoporosis, as they emerged as the less sensitized category. Women on AET for breast cancer represent a great matter of concern, since they frequently have reduced BMD and a history of fractures already at first observation. Despite this, they have low dietary calcium intake, even though the total calcium intake was not evaluated in our study. This finding could be related to discouragement in consuming dairy products, the main source of calcium, in clinical practice. However, it must be considered a hypothesis, as our study did not evaluate the motivations behind the dietary habits. Individualized nutritional assessment and counseling to meet calcium requirements through appropriate dietary sources, coordinated with oncology and bone-health care, appears essential in this population.

## Figures and Tables

**Table 1:** General characteristics of study population, both total and sex-stratified. Categorical variables were expressed as frequencies (percentages). Continuous variables are expressed as means ± SD.

Parameters	Total (n = 132)	Male (n = 10)	Female (n = 122)	*p* Value
Age (years; n = 132)	65 ± 10	65 ± 9	66 ± 10	0.8
BMI (kg/m^2^; n = 132)	24.9 ± 4.5	26.6 ± 5	24.8 ± 4.5	0.1
Previous fragility fractures (%):				0.9
- No fractures	59 (44.8%)	4 (40%)	55 (45.1%)	
- Minor fractures	18 (13.6%)	1 (10%)	17 (13.9%)	
- Vertebral fractures	42 (31.8%)	4 (40%)	38 (31.1%)	
- Femoral fractures	13 (9.8%)	1 (10%)	12 (9.8%)	
Treatment (%):				0.3
- No treatment or vitamin D supplementation only	55 (41.7%)	4 (40%)	51 (41.8%)	
- Bisphosphonates	48 (36.4%)	5 (50%)	43 (35.2%)	
- Denosumab	23 (17.4%)	1 (10%)	22 (18%)	
- Teriparatide	6 (4.5%)	0%	6 (4.9%)	
Oral calcium supplementation (yes, %)	19 (14.4%)	4 (40%)	15 (12.3%)	0.04
Blood calcium (mg/dL; n = 66)	9.2 ± 0.5	9.1 ± 0.3	9.2 ± 0.5	0.5
Blood phosphorus (mg/dL; n = 42)	3.3 ± 0.6	3 ± 0.7	3.4 ± 0.5	0.2
25(OH)D (ng/mL; n = 44)	33 ± 13	33 ± 17	33 ± 13	0.8
PTH (pg/mL; n = 41)	67 ± 46	50 ± 8.6	68 ± 48	0.5
CTX (pg/mL; n = 46)	344 ± 231	277 ± 131	353 ± 240	0.5
bALP (mcg/L; n = 44)	10.5 ± 5	7 ± 3.4	10.8 ± 5.1	0.2
BMD L1–L4 (g/cm^2^; n = 132)	1.002 ± 0.187	1.02 ± 0.17	1.001 ± 0.189	0.9
BMD femoral neck (g/cm^2^; n = 82)	0.769 ± 0.08	0.868 ± 0.1	0.757 ± 0.09	0.006
BMD total hip (g/cm^2^; n = 82)	0.830 ± 0.116	0.943 ± 0.13	0.815 ± 0.11	0.009
T-score L1–L4 (SD; n = 132)	−1.5 ± 1.5	−1.4 ± 1.4	−1.5 ± 1.6	0.9
T-score femoral neck (SD; n = 124)	−1.6 ± 1	−1.3 ± 0.8	−1.7 ± 0.9	0.09
T-score total hip (SD; n = 126)	−1.7 ± 0.9	−1.4 ± 0.9	−1.7 ± 0.9	0.2
TBS L1–L4 (n = 132)	1.15 ± 0.15	1.14 ± 0.14	1.15 ± 0.15	0.9
Patients with T-score < −2.5 in at least one site (%)	48 (36.4%)	4 (40%)	44 (36.1%)	0.4
Subgroup category (%):				0.3
- Postmenopausal	21 (15.9%)	0%	21 (17.2%)	
- Secondary osteoporosis	54 (40.9%)	6 (60%)	48 (39.3%)	
- Severe osteoporosis	57 (43.2%)	4 (40%)	53 (43.5%)	

BMI: body mass index; 25(OH)D: 25-OH-cholecalciferol; PTH: parathyroid hormone; CTX: C-terminal telopeptide of type 1 collagen; bALP: bone alkaline phosphatase; BMD: bone mineral density; SD: standard deviation; TBS: trabecular bone score. *p* values refer to the comparison between male and female patients.

**Table 2:** Daily dietary calcium intake in the female population stratified by age tertiles, presence and etiology of osteoporosis, lumbar and femoral T-score, TBS L1–L4, presence of fractures and pharmacological treatment.

Category (Female Population)	Daily Dietary Calcium Intake (mg)	*p* Value
Age		0.334
- 1st tertile (43–57 years; n = 32)	872 ± 415	
- 2nd tertile (58–74 years; n = 63)	984 ± 403	
- 3rd tertile (75–90 years; n = 27)	1014 ± 358	
Diagnosis		0.193
- Normal BMD (n = 6)	631 ± 131	
- Osteopenia (n = 73)	954 ± 419	
- Osteoporosis (n = 43)	1005 ± 365	
Subgroup		0.379
- Postmenopausal (n = 21)	976 ± 410	
- Severe (n = 53)	1011 ± 420	
- Secondary (n = 48)	900 ± 364	
T-score L1–L4		0.547
- −1 SD (n = 41)	977 ± 412	
- Between −1 and −2.5 SD (n = 54)	921 ± 380	
- <−2.5 SD (n = 27)	1021 ± 415	
T-score total hip		0.316
- −1 SD (n = 20)	822 ± 293	
- Between −1 and −2.5 SD (n = 80)	972 ± 444	
- <−2.5 SD (n = 22)	990 ± 260	
T-score femoral neck		0.554
- −1 SD (n = 26)	983 ± 446	
- Between −1 and −2.5 SD (n = 68)	912 ± 403	
- <−2.5 SD (n = 28)	1005 ± 340	
TBS L1–L4		0.519
- Normal (n = 7)	842 ± 251	
- Partially degraded (n = 42)	922 ± 394	
- Degraded (n = 73)	993 ± 408	
Fractures		0.156
- No fractures (n = 55)	906 ± 361	
- Minor fractures (n = 17)	1151 ± 527	
- Vertebral fractures (n = 38)	939 ± 322	
- Hip fractures (n = 12)	1032 ± 525	
Treatment		0.005 (post hoc analysis: teriparatide vs. no treatment: *p* = 0.014; teriparatide vs. only vitamin D oral supplementation: *p* = 0.032; teriparatide vs. bisphosphonates: *p* = 0.021; teriparatide vs. denosumab: *p* = 0.003)
- No treatment (n = 7)	726 ± 230	
- Only vitamin D oral supplements (n = 44)	983 ± 403	
- Bisphosphonates (n = 43)	959 ± 374	
- Denosumab (n = 22)	834 ± 314	
- Teriparatide (n = 6)	1480 ± 526	

SD: standard deviation; TBS: trabecular bone score.

**Table 3:** General characteristics of BC subgroup, compared with postmenopausal women and women with severe osteoporosis. Categorical variables were expressed as frequencies (percentages). Continuous variables are expressed as means ± SD.

Parameters (Female Population)	Breast Cancer (n = 35)	Postmenopausal (n = 21)	Severe Osteoporosis (n = 53)	*p* Value
Age (years; n = 109)	60 ± 10	64 ± 9	71 ± 9	<0.005 (post hoc analysis: severe osteoporosis vs. postmenopausal: *p* = 0.012; severe osteoporosis vs. breast cancer: *p* < 0.005)
BMI (kg/m^2^; n = 109)	25 ± 5	25 ± 5	25 ± 4	0.9
Previous fragility fractures (%):				<0.005 (post hoc analysis: severe osteoporosis vs. postmenopausal: *p* < 0.005; severe osteoporosis vs. breast cancer: *p* < 0.005)
- No fractures	26 (74.3%)	21 (100%)	0%	
- Minor fractures	5 (14.3%)	0%	7 (13.2%)	
- Vertebral fractures	1 (2.9%)	0%	37 (69.8%)	
- Femoral fractures	3 (8.6%)	0%	9 (17%)	
Treatment (%):				<0.005 (post hoc analysis: severe osteoporosis vs. postmenopausal: *p* < 0.005; severe osteoporosis vs. breast cancer: *p* < 0.005)
- No treatment or vitamin D supplementation only	19 (54.3%)	14 (66.6%)	10 (18.9%)	
- Bisphosphonates	14 (40%)	7 (33.4%)	18 (34%)	
- Denosumab	2 (5.7%)	0%	19 (35.8%)	
- Teriparatide	0%	0%	6 (11.3%)	
Oral calcium supplementation (yes, %)	1 (2.9%)	4 (19%)	9 (17%)	0.07
Daily dietary calcium intake (mg; n = 109)	854 ± 345	976 ± 411	1111 ± 420	0.2
Blood calcium (mg/dL; n = 50)	9 ± 0.4	9.2 ± 0.5	9.3 ± 0.4	0.06
Blood phosphorus (mg/dL; n = 27)	3 ± 0.3	3.4 ± 0.6	3.5 ± 0.6	0.5
25(OH)D (ng/mL; n = 34)	33 ± 16	34 ± 18	35 ± 10	0.9
PTH (pg/mL; n = 28)	59 ± 22	67 ± 35	65 ± 53	0.9
CTX (pg/mL; n = 34)	338 ± 208	417 ± 225	280 ± 215	0.3
bALP (mcg/L; n = 29)	8.4 ± 3.3	9.6 ± 3.5	10.8 ± 4.8	0.5
BMD L1–L4 (g/cm^2^; n = 109)	0.985 ± 0.136	1.067 ± 0.236	0.974 ± 0.187	0.2
BMD femoral neck (g/cm^2^; n = 65)	0.776 ± 0.104	0.746 ± 0.072	0.743 ± 0.082	0.7
BMD total hip (g/cm^2^; n = 65)	0.827 ± 0.143	0.806 ± 0.049	0.805 ± 0.108	0.7
T-score L1–L4 (SD; n = 109)	−1.7 ± 1.1	−1 ± 1.9	−1.7 ± 1.6	0.2
T-score femoral neck (SD; n = 96)	−1.4 ± 0.9	−1.6 ± 0.5	−1.8 ± 1	0.5
T-score total hip (SD; n = 98)	−1.4 ± 1.1	−1.6 ± 0.5	−1.9 ± 0.8	0.5
TBS L1–L4 (n = 109)	1.22 ± 0.13	1.16 ± 0.15	1.10 ± 0.15	0.2
Patients with T-score < −2.5 in at least one site (%)	7 (20%)	6 (28.6%)	27 (50.9%)	0.05 (post hoc analysis: severe osteoporosis vs. postmenopausal: *p* = 0.174; severe osteoporosis vs. breast cancer: *p* < 0.095)

BMI: body mass index; 25(OH)D: 25-OH-cholecalciferol; PTH: parathyroid hormone; CTX: C-terminal telopeptide of type 1 collagen; bALP: bone alkaline phosphatase; BMD: bone mineral density; SD: standard deviation; TBS: trabecular bone score. Thirteen women with secondary osteoporosis not related to AET were excluded from the analysis.

**Table 4:** Daily dietary calcium intake among women with BC, stratified by age tertiles, diagnosis of osteoporosis, lumbar and femoral T-score, TBS L1–L4, presence of fractures and pharmacological treatment.

Category	Daily Dietary Calcium Intake (mg)	*p* Value
Age		0.1
- 1st tertile (43–57 years; n = 6)	710 ± 311	
- 2nd tertile (58–74 years; n = 21)	849 ± 365	
- 3rd tertile (75–90 years; n = 8)	1025 ± 296	
Diagnosis		0.09
- Normal BMD (n = 0)	No participants	
- Osteopenia (n = 28)	805 ± 331	
- Osteoporosis (n = 7)	1051 ± 349	
T-score L1–L4		0.2
- −1 SD (n = 11)	778 ± 368	
- Between −1 and −2.5 SD (n = 18)	830 ± 308	
- <−2.5 SD (n = 6)	1069 ± 379	
T-score total hip		0.3
- −1 SD (n = 7)	641 ± 283	
- Between −1 and −2.5 SD (n = 23)	861 ± 353	
- <−2.5 SD (n = 5)	989 ± 200	
T-score femoral neck		0.8
- −1 SD (n = 9)	741 ± 245	
- Between −1 and −2.5 SD (n = 21)	825 ± 372	
- <−2.5 SD (n = 5)	904 ± 77	
TBS L1–L4		0.8
- Normal (n = 3)	768 ± 286	
- Partially degraded (n = 17)	832 ± 357	
- Degraded (n = 15)	897 ± 357	
Fractures		0.9
- No fractures (n = 26)	848 ± 351	
- Minor fractures (n = 5)	912 ± 326	
- Hip fractures (n = 3)	784 ± 496	
Treatment		0.5
- No treatment (n = 3)	746 ± 323	
- Only vitamin D oral supplements (n = 16)	867 ± 312	
- Bisphosphonates (n = 14)	907 ± 401	

The category “fractures” does not include “vertebral fractures” as only one patient reported a vertebral fracture and then was excluded from the analysis. The category “treatment” does not include “denosumab” as only two patients took this drug and then were excluded from the analysis. SD: standard deviation; TBS: trabecular bone score.

## Data Availability

The data used to support the findings of the study are available from the corresponding author upon request.

## Abbreviations

The following abbreviations are used in this manuscript:

AET	Adjuvant Endocrine Therapy
ER	Estrogen Receptor
BMI	Body Mass Index
IOF	International Osteoporosis Foundation
PTH	Parathyroid Hormone
bALP	Bone Alkaline Phosphatase
BC	Breast Cancer
SIOMMMS	Italian Society for Osteoporosis, Mineral Metabolism and Skeletal Diseases
BMD	Bone Mineral Density
25(OH)D	25-OH-Cholecalciferol
CTX	C-Terminal Telopeptide of Type 1 Collagen
DXA	Dual-Energy X-Ray Absorptiometry
SD	Standard Deviation
TBS	Trabecular Bone Score
VFA	Vertebral Fracture Assessment
GnRH	Gonadotropin-Releasing Hormone
AI	Aromatase Inhibitor
SERMs	Selective Estrogen Receptor Modulators
